# Expression and enhancement of FABP4 in septoclasts of the growth plate in FABP5-deficient mouse tibiae

**DOI:** 10.1007/s00418-020-01953-y

**Published:** 2021-01-04

**Authors:** Yasuhiko Bando, Nobuko Tokuda, Yudai Ogasawara, Go Onozawa, Arata Nagasaka, Koji Sakiyama, Yuji Owada, Osamu Amano

**Affiliations:** 1 Division of Anatomy, Meikai University School of Dentistry, 1-1 Keyakidai, Sakado, Saitama 3500283 Japan; 2 Department of Anatomy (Macro), School of Medicine, Dokkyo Medical University, 880 Kitakobayashi, Mibu, Tochigi 3210293 Japan; 3 Division of Maxillofacial Surgery II, Meikai University School of Dentistry, 1-1 Keyakidai, Sakado, Saitama 3500283 Japan; 4 Division of Maxillofacial Surgery I, Meikai University School of Dentistry, 1-1 Keyakidai, Sakado, Saitama 3500283 Japan; 5 Department of Organ Anatomy, Tohoku University Graduate School of Medicine, 2-1 Seiryo-machi, Aoba-ku, Sendai, Miyagi 9808575 Japan

**Keywords:** Septoclasts, FABP4, FABP5, Growth plate, PPARγ, Mouse

## Abstract

In our previous study, fatty acid-binding protein 5 (FABP5) was expressed in septoclasts with long processes which are considered to resorb uncalcified matrix of the growth plate (GP) cartilage, and no apparent abnormalities were detected in the histo-architecture of the GP of FABP5-deficient (FABP5^−/−^) mice. Those finding lead us to hypothesize that another FABP can compensate the deletion of FABP5 in septoclasts of its gene-mutant mice. Based on the hypothesis, the present study examined the expression levels of several other FABPs in septoclasts and their morphology in FABP5^−/−^ mouse tibiae. Processes of FABP5^−/−^ septoclasts tend to be shorter than wild septoclasts. FABP4-positive septoclasts in FABP5^−/−^ mice were more numerous than those cells in wild mice.

Peroxisome proliferator-activated receptor (PPAR) γ was expressed in FABP4-positive septoclasts of FABP5^−/−^ mice as well as mice administered with GW1929, a PPARγ agonist, suggesting that the occurrence of PPARγ induces an increase of FABP4-positive septoclasts. The present finding suggests that the functional exertion of FABP5 in septoclasts is supplemented by FABP4 in normal and FABP5^−/−^ mice, and that the expression of FABP4 is up-regulated in accompany with PPARγ in FABP5^−/−^ for maintenance of resorptive activity in the GP.

**Supplementary Information:**

The online version contains supplementary material available at 10.1007/s00418-020-01953-y.

## Introduction

Bone development is carried out by membranous or endochondral ossification. Endochondral ossification participates continuously in pre- and post-natal bone growth in the epiphyseal growth plate (GP) of long bones. Chondrocytes in the GP are surrounded by longitudinal and transverse septa consisting of calcified and uncalcified matrices, respectively (Schenk et al.^1967^; Lee et al. 1995; Amizuka et al. 2012). Resorption of the GP cartilage is principally performed by osteoclasts (Nakamura and Ozawa 1996; Mackie et al. 2011), and osteoclasts resorb the longitudinal septa made of calcified cartilages (Savostin-Asling and Asling 1975). Osteoclasts, or more correctly termed chondroclasts in case of involvement in the cartilage resorption, are located on the longitudinal septa a short distance away from the invading blood vessels (Nakamura and Ozawa 1996); therefore, to resorb the hypertrophic zone of the GP, the transverse septum facing the bone marrow cavity is initially broken to expose the cartilage lacunae and upper portions of the longitudinal septa.

Septoclasts are located along the chondro-osseous junction (COJ) of the GP and adjacent to growing capillaries of invading vessels, and the cells are, therefore, previously termed perivascular cells (Schenk et al.^1967^). They produce proteinases such as cathepsin B (Lee et al. 1995) and MMP-13 (Nakamura et al. 2004) which resolve the collagenous substrate. Apexes of the long processes from the septoclastic bodies are attached to the uncalcified transverse septa, and therefore, septoclasts are considered to be involved in the cleavage of the transverse septa of the GP (Schenk et al.^1967^; Lee et al. 1995; Odgren et al. 2016). A shortening of septoclastic processes and a decrease in their number lead septoclasts to diminish the mechanical linkage between the cells and the transverse septa, resulting in the reduction of the cleavage of the transverse septa in the GP (Bando et al. 2014, 2017).

Fatty acid-binding proteins (FABPs) comprise a multigene family of intracellular lipid binding proteins, and they are involved in the promotion of the cellular uptake and transport of fatty acids, the targeting of fatty acids to specific metabolic pathways, and the regulation of gene expression (Owada 2008; Storch and Thumser 2010; Smathers and Petersen 2011). We previously revealed the exclusive expression of FABP5, epidermal-type FABP, in septoclasts along the COJ (Bando et al. 2014). FABP5 was suggested to mediate the transport of long chain fatty acid to mitochondria for energy metabolism in septoclasts (Bando et al. 2014) and the transport of retinoic acid to nuclei for induction of signaling pathways mediated by PPARβ/δ, resulting in maintenance of proliferation and morphology of septoclasts (Bando et al. 2017).

There has so far been no clear evidence for morphological abnormalities in any tissues of FABP5-deficient (FABP5^−/−^) mice (Owada et al. 2002a), although various functional phenotypes of FABP5^−/−^ mice have been clarified in water permeability of the skin (Owada et al. 2002b), keratinocyte differentiation (Ogawa et al. 2011), and anti-inflammatory response of macrophage (Moore et al. 2015). There have been data indicating that the gene-deletion of a FABP subtype is compensated by an increased expression of another FABP subtype (Owada et al. 2002b).

The expression of multiple FABP subtypes in a single cell species has also been reported, such as FABP4 and FABP5 in adipocytes and macrophages (Furuhashi et al. 2014; Hotamisligil and Bernlohr 2015). According to their studies, both FABP4 and FABP5 were synergistically involved in the development of insulin resistance and atherosclerosis. FABP4-deficiency in adipocytes is compensated by enhancement of FABP5 expression (Shaughnessy et al. 2000); however, compensation by other types of FABP does not occur in macrophages in FABP4-deficiency (Makowski et al. 2001) or adipocytes in FABP5-deficiency (Maeda et al. 2003). The transcriptional activity is enhanced in the nucleus by PPARγ bound to FABP4, resulting in the induction of adipogenesis and differentiation of macrophages from monocytes (Tontonoz et al. 1994, 1998; Tan et al. 2002; Adida and Spener 2006).

We hypothesized that a possible reduction in the activity of cartilage resorption by septoclasts owing to FABP5-deficiency is compensated by renewed expression of alternative type(s) of FABP. In the present study, to clarify the hypothesis, we investigated the expression levels of several subtypes of FABP in septoclasts and evaluated the quantity and morphology of septoclasts in the tibia GP of FABP5^−/−^ mice.

## Materials and methods

### Animals

C57BL/6 wild-type (WT) mice and FABP5^−/−^ mice (on the C57BL/6 background, Owada et al. 2002b; Tokuda et al. 2010) aged 3 weeks were used. C57BL/6 WT mice were purchased from Sankyo Labo Service (Tokyo, Japan).

### Treatment with GW1929

Mice treated with GW1929, the selective peroxisome proliferator-activated receptor (PPARγ) ligand (Brown et al. 1999), were used according to previous studies (Lenhard et al. 1999; Moore-Carrasco et al. 2008; Tickner et al. 2011). C57BL/6 WT mice aged 3 weeks (*n* = 4) were fed ad libitum with GW1929 (Cayman chemical, Ann Arbor, MI, USA) (10 mg/kg body weight diluted in 4% dimethyl sulfoxide (DMSO)/water per a day) by oral gavage for 2 weeks, while vehicle (4% DMSO/water) was administrated to control mice (*n* = 4).

### Tissue preparation

Mice were administered with pentobarbital sodium (70 mg/kg body weight) and perfused through the heart first with saline and subsequently with 4% paraformaldehyde in 0.1 M phosphate buffer (pH 7.4). Then the tibiae were dissected and immersed in the same fixative overnight at 4 °C. For preparation of specimen for skeletal staining and measurement or micro-computed tomography (μCT) analysis, after muscle and connective tissue were removed, tibiae were stored in the water at 4 °C. For preparation of specimen for histochemistry, tibiae were subsequently decalcified in 10% ethylenediaminetetraacetic acid (EDTA, pH 7.2) for 20 days at 4 °C. Specimens were then immersed overnight in 30% sucrose in 0.1 M phosphate buffer. Proximal tibiae were dissected out from the knee joints, and frozen sagittal sections of 20 μm or 10 μm thickness, for three dimensional (3D) analysis or for all other analyses, respectively, were cut on a cryostat.

### Image acquisition

The light microscopic observations of histological or immunohistochemical staining were made by use of a BX50 microscopy (Olympus, Tokyo, Japan) equipped with an Axiocam ERc 5 s camera (Carl Zeiss, Obercohen, Germany) and an SE64 Rel.4.9.1. SP2 software (Carl Zeiss). The objective lenses used were UPlanApo 10x/0.40 and UPlanApo 20x/0.70 (Olympus). The observations of immunofluorescent staining were made by use of a confocal laser-scanning microscope (LSM800, Carl Zeiss) equipped with a Zen 2.1 [blue edition] software (Carl Zeiss). The objective lens used was Plan-Apochromat 20x/0.8 M27.

### Morphometry and histology

To compare the morphology between WT mice and FABP5^−/−^ mouse tibiae, we performed a morphometrical analysis. Measurement of mineralized bone length of the tibiae: Procedure of Alizarin red and Alcian blue was established previously (McLeod 1980). Six tibiae collected from 6 mice for each group were stained in a solution 0.1% Alizarin red, 0.3% Alcian blue, glacial acetic acid and 70% ethanol at a volumetric ratio of 1:1:1:17 for 2–3 days. Stained tibiae were stored in 1% KOH in aqueous solution of 20% glycerin for 2 days and transferred to aqueous solution of 20%, 50%, 80% glycerin and stored in 100% glycerin. Alizarin red-stained mineralized bone lengths of the tibiae were measured.

Analysis of the morphology of trabecular just under the COJ: Five tibiae collected from 5 mice for each group were scanned using μCT (Skyscan 1176, Bruker, Billerica, MA, USA) and morphometric parameters of proximal tibial trabeculae [trabecular number (Tb.N), trabecular thickness (Tb.Th) and trabecular separation (Tb.Sp)] were analyzed by host software (Bruker, Billerica, MA, USA).

Measurement of the height of the GP and the number of vacated cartilage lacunae: Midsagittal sections of the five proximal tibiae collected from 5 mice for each group were stained routinely with H–E (hematoxylin and eosin). The height was measured in the middle of the GP, and fifty sections for each group were measured and analyzed statistically. The number of vacated cartilage lacunae was measured by taking an average of the number of cartilage lacunae which lose their distinctive cytoplasmic features in unit squares with 200 μm × 1000 μm along the line of the COJ. Ten squares for each group were measured and analyzed statistically.

### Immunohistochemistry

Immunohistochemistry was performed as described in our previous study (Bando et al. 2014). Briefly, the cryosections on glass slides were treated with 0.3% Triton X-100 in phosphate-buffered saline (PBS) for 60 min, 0.3% H_2_O_2_/methanol for 10 min, 10% fetal bovine serum (FBS, Nichirei, Tokyo, Japan) in 0.1 M PBS for 60 min and incubated with rabbit anti-rat FABP5 polyclonal antibody (0.5 μg/ml, Owada et al. 2001) or rabbit anti-mouse FABP3 (heart-type FABP) polyclonal antibody (0.5 μg/ml, Islam et al. 2014) or rabbit anti-mouse FABP7 (brain-type FABP) polyclonal antibody (0.5 μg/ml, Tokuda et al. 2010) or goat anti-human cathepsin B (AF965; 10 μg/ml, R&D Systems, Minneapolis, MN, USA) or goat anti-mouse FABP4 (adipocyte-type FABP, AF1443; 10 μg/ml, R&D Systems) or goat anti-rat FABP1 (liver-type FABP, AF1565; 10 μg/ml, R&D Systems) or goat anti-rat FABP2 (intestine-type FABP, AF1565; 10 μg/ml, R&D Systems) in PBS overnight at room temperature. They were incubated with the secondary antibody, i.e., biotin-conjugated goat anti-rabbit IgG (426,012; Nichirei) or rabbit anti-goat IgG (416,022; Nichirei) for 60 min and reacted with horseradish peroxidase (HRP)-conjugated streptavidin (Nichirei) for 45 min. 3,3′-diaminobenzidine tetrahydrochloride (DAB, Dojindo, Kumamoto, Japan) was applied to visualize sites of the antigen–antibody reaction. Immunostaining for FABP1, 2, 3, 4 and 7 were applied for screening of an alternative expression of other types of FABP rather than FABP5 in septoclasts.

For double immunofluorescent staining, sections were treated overnight at room temperature with a mixture of rabbit anti-FABP4 (12,802–1-AP; 1:50, Proteintech, Chicago, IL, USA) and goat anti-mouse CD31 (AF3628; 10 μg/ml; R&D system), or rabbit anti-FABP4 (12,802–1-AP; 1:50, Proteintech) and goat anti-mouse PDGFRβ (AF1042; 10 μg/ml; R&D system), or rabbit anti-FABP4 (12,802–1-AP; 1:50, Proteintech) and goat anti-human cathepsin B (AF965; 10 μg/ml, R&D Systems), or rabbit anti-rat FABP5 (0.5 μg/ml, Owada et al. 2001) and goat anti-mouse FABP4 (AF1443; 10 μg/ml, R&D Systems), or rabbit anti-human PPARγ (MA5-14,889; 1:50, Invitrogen, Carlsbad, CA, USA) and goat anti-mouse FABP4 (AF1443; 10 μg/ml, R&D Systems). After having been washed with PBS, the sections were treated for 60 min with a mixture of Cy3-conjugated donkey anti-rabbit IgG (AP182C; 1:200; Merck Millipore, Billerica, MA, USA) plus FITC-conjugated donkey anti-goat IgG (AP180F; 1:200; Merck Millipore). For double immunofluorescent staining for PPARγ and FABP4, sections were subsequently treated with 4,6-diamidino-2-phenylindole dihydrochloride (DAPI, ImmunoBioScience, Mukilteo, WA, USA) for nuclear staining. 

### 3D analysis

Sections of 20 μm thickness were stained in immunofluorescence with goat anti-human cathepsin B (AF965; 10 μg/ml, R&D Systems) or anti-mouse FABP4 (AF1443; 10 μg/ml, R&D Systems) and Cy3-conjugated donkey anti-goat IgG (AP180C; 1:200, Merck Millipore), or rabbit anti-rat FABP5 (0.5 μg/ml, Owada et al. 2001) and Cy3-conjugated donkey anti-rabbit IgG (AP182C; 1:200; Merck Millipore). Sections were subsequently treated with 4,6-diamidino-2-phenylindole dihydrochloride (DAPI, ImmunoBioScience) for nuclear staining. 3D images and movies were reconstructed by use of Zen 2.1 [blue edition] software (Carl Zeiss).

### Measurement of length of processes

The length of processes of septoclasts are shown in Figs. 2f, h and 3c, f, i. Midsagittal sections of the eight proximal tibiae collected from 8 WT or FABP5^−/−^ mice were used for immunofluorescent staining for cathepsin B, FABP4, or FABP5. Sixteen septoclasts were randomly selected from 3D reconstructed images of each group, and the length of process of them were measured using Image J (The Research Services Branch, National Institute of Mental Health, Bethesda, MD, USA) and analyzed statistically.

#### Electron microscopy

After perfusion with 4% paraformaldehyde in 0.1 M phosphate buffer, tibiae were dissected and immersed in the same fixative overnight at 4 °C, and for preparation of specimen for electron microscopy, tibiae were immersed in 2% glutaraldehyde in 0.1 M cacodylate buffer (pH 7.4) for 2 h at 4 °C and postfixed with 1% OsO_4_ for 2 h at 4 °C. Specimen were dehydrated in a graded ethanol series, and embedded in araldite M resin (Nisshin EM, Tokyo, Japan). Ultrathin sections of tibiae were prepared using ultramicrotome. Ultrathin sections were stained with uranyl acetate and lead citrate, and observed under a JEM-1210 transmission electron microscope (JEOL, Tokyo, Japan).

#### Cell count

Measurement of number of immunopositive cells per areas was according to our previous study (Bando et al. 2014; 2017) by taking an average of the number of immunopositive cell bodies in unit squares with 200 μm × 1000 μm along the line of the COJ of proximal tibiae. In the present study, a cell with a clearly observable cell body containing a nucleus was counted as a cell. The number of septoclasts immunopositive to FABP5 and/or FABP4 were measured in 10 WT or FABP5^−/−^ mice. The number of septoclasts immunopositive to FABP5 and/or FABP4 were measured in 4 control or GW1929-treated mice. Twenty squares for each group were measured and analyzed statistically.

### Statistical analysis

The cell counts, morphometrical and histological measurements were expressed as the mean ± standard deviation in graphs, and they were treated by means of Mann–Whitney *U* test. *P* values < 0.05 were regarded as statistically significant.

## Results

### Phenotype of the tibia of FABP5-/- mice

**Fig. 1:**
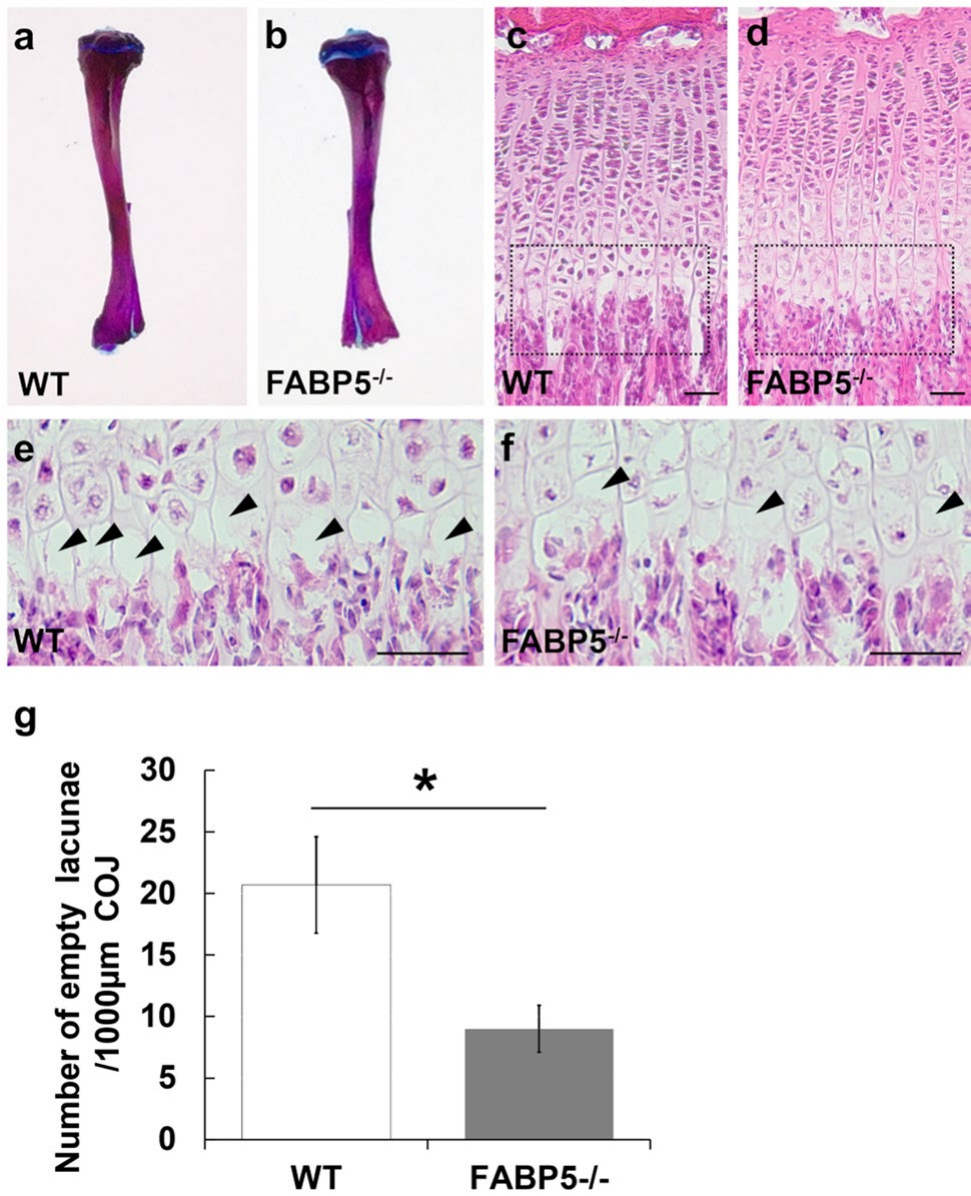
Phenotypes of the tibiae of wild-type (WT) and FABP5-deficient (FABP5^−/−^) mice in appearance (**a**, **b**), sagittal sections of the growth plate (GP) (**c**, **d**), higher-magnified images of dotted square region in c and d, respectively (**e**, **f**), and the graph showing number of empty lacunae lacking chondrocytes (**g**). Arrowheads: empty lacunae (**e**, **f**). Scale bars: 50 μm (**c**–**f**). **g** Mean SD; * P < 0.001. [n = 10 (**g**)]. Note that the hypertrophied lacunae including chondrocytes along the chondro-osseous junction (COJ) in FABP5^−/−^ mice increased significantly compared to WT

No significant differences between tibiae of FABP5^−/−^ mice and those of WT mice were seen in macroscopic observation (Fig. 1a, b) as well as bone length, number and thickness of the trabeculae, pattern of trabecular separation and the GP thickness measured by micro CT or morphometrical analyses (data not shown). Although no apparent difference was found in whole sagittal section of the GP (Fig. 1c, d), a notable difference was found in the last cartilaginous lacunae just below the COJ, in which hypertrophic chondrocytes were often contained in the FABP5^−/−^ mice (Fig. 1f) in contrast to infrequent occurrence of chondrocytes in WT mice (Fig. 1e). Consequently, a significant difference in the number of empty lacunae per unit area of the COJ of the GP was detected statistically between FABP5^−/−^ and WT tibiae (Fig. 1g).

### Septoclasts in FABP5^−/−^ tibiae

**Fig. 2:**
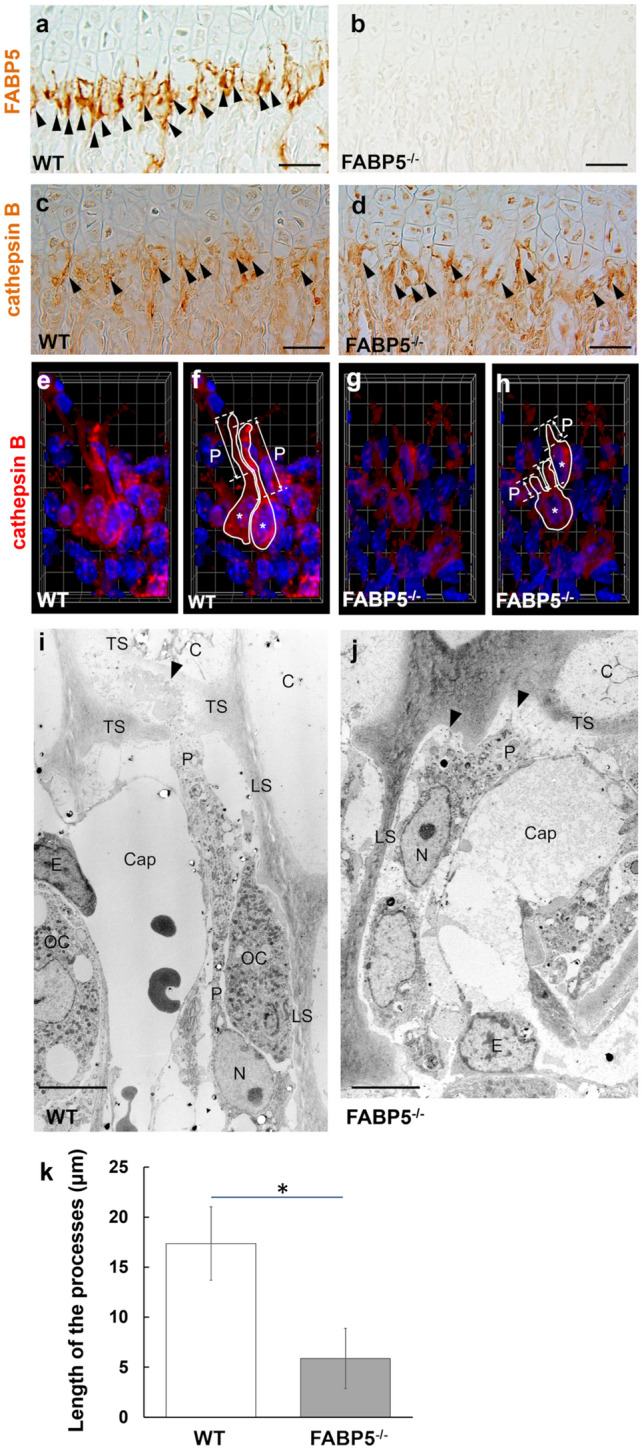
Septoclasts at the COJ of proximal tibiae of WT and FABP5^−/−^ mice. Light micrographs of longitudinal section stained for marker of septoclasts, FABP5 (**a**, **b**) and cathepsin B (**c**, **d**). Three-dimensional (3D) reconstructed images of septoclasts stained for cathepsin B (**e**–**h**). Electron micrographs of septoclasts at the COJ (**i**, **j**). The graph showing length of the septoclastic processes (**k**). **a**, **c**, **d** Arrowheads: immunopositive cells. **f**, **h** Images drawing the outline of septoclasts as same as e and g, respectively. Asterisks: cell bodies of septoclasts, Dotted lines: boundaries of septoclastic processes, Solid lines: outline of septoclastic body and processes, *P* length of process. **i**, **j** Arrowheads: tips of the processes of septoclasts, *P* processes of septoclasts, *N* nucleus of septoclast, *C* chondrocyte, *LS* longitudinal septum, *TS* transverse septum, *Cap* capillary, *E* endothelial cell, *OC* osteoclast. **k** Mean SD, *P < 0.001 [*n* = 16]. Scale bars: 50 μm (**a**–**d**), 6.4 μm (length of the side of a square in 3D grid, **e**–**h**), 5 μm (**i**, **j**)

As expected, FABP5-immunoreactivity was not seen in FABP5^−/−^ tibiae (Fig. 2b). Septoclasts of FABP5^−/−^ tibiae, which were identified by immunoreactivity for cathepsin B as their known marker (Lee et al. 1995), were localized closer to the COJ than those of WT tibiae immunopositive for FABP5 and cathepsin B (Fig. 2a, c, and d).

In 3D-reconstruction by a confocal laser scanning microscope, although most septoclasts, immunostained for cathepsin B, had long processes extending from the cell bodies towards the GP cartilage in WT tibiae (Fig. 2e, f, movies shown in Supplementary data S1, S2), septoclasts having much shorter processes were dominant in FABP5^−/−^ tibiae (Fig. 2g, h).

In electron microscopy, reflecting the light microscopic findings described above, septoclasts in FABP5^−/−^ tibiae were located closer to the transverse septa and their thicker and shorter processes protruded from their spherical cell bodies (Fig. 2j), in contrast to thin and long processes of wild septoclasts extending between the longitudinal septum and the capillary endothelial cell with their apex attaching to the transverse septum (Fig. 2i).

In morphometrical analysis, the length of septoclastic processes in FABP5^−/−^ mice was significantly shorter compared to that in WT ones (Fig. 2k).

### Occurrence of FABP4-immunoeacitivity in WT and FABP5-/- mice

Immunopositive staining for FABP4 was found along the COJ of the GP, whereas no specific reaction was found for FABP1, 2, 3 and 7 in septoclasts (data not shown).

**Fig. 3:**
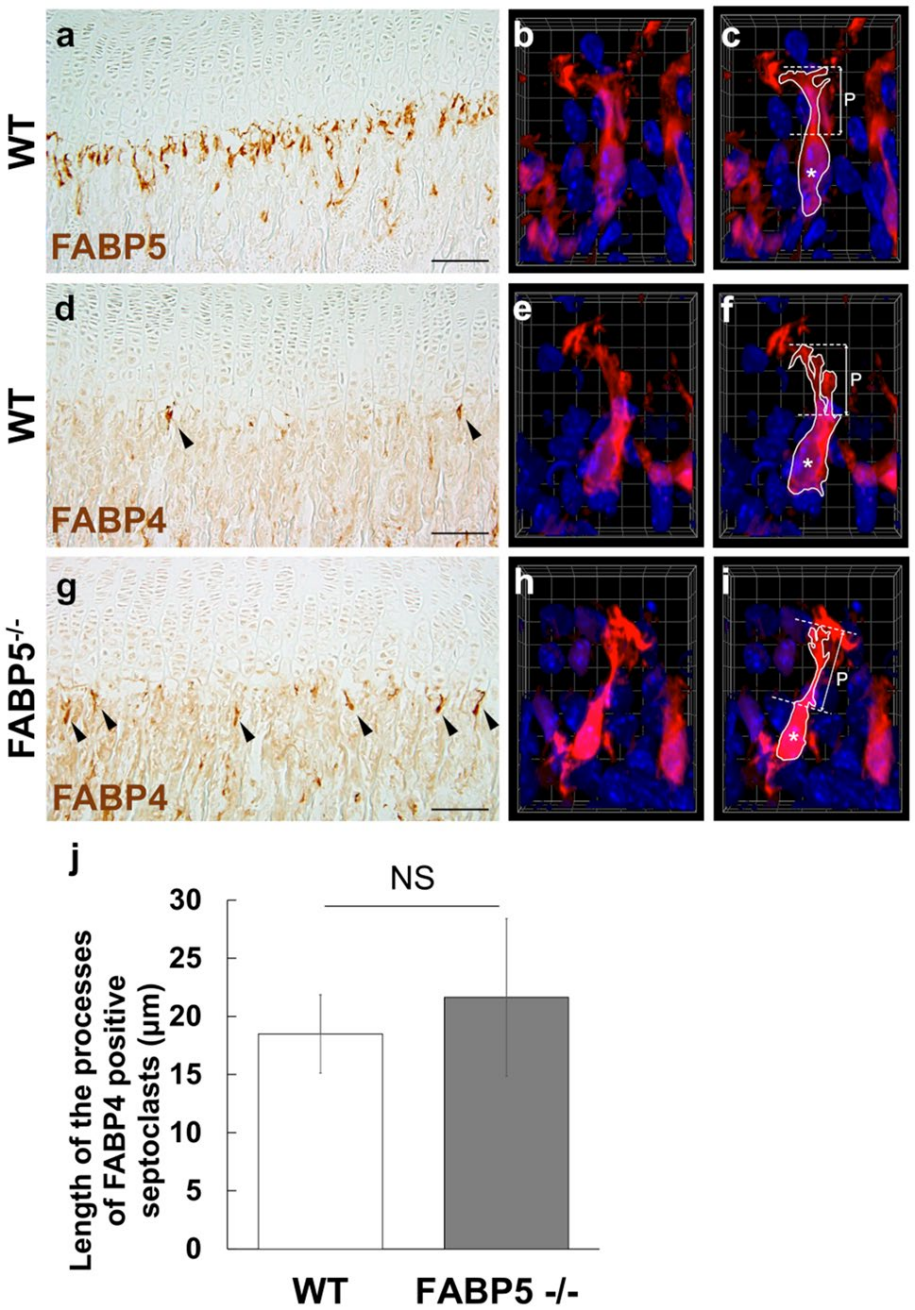
FABP4-immnopositive cells in the chondro-osseous junction (COJ) of WT and FABP5^−/−^ mice. Light micrograph (**a**) and 3D-reconstructed image (**b**, **c**) of FABP5-immunopositive septoclasts in WT mice. Light micrograph (**d**) and 3D-reconstructed image (**e**, **f**) of FABP4-immunopositive cells in WT mice. Light micrograph (**g**) and 3D-reconstructed image (**h**, **i**) of FABP4-immunopositive cells in FABP5^−/−^ mice. **d**, **g** Arrowheads: FABP4 immunopositive cells. **c**, **f**, **i** Images drawing the outline of septoclasts as same as (**b**, **e** and **h**), respectively. Asterisks: cell bodies of septoclasts, Dotted lines: boundaries of septoclastic processes, Solid lines: outline of septoclastic body and processes, *P* length of process. **j** Mean SD, [*n* = 16]. *NS* no significance. Scale bars: 100 μm (**a**, **d**, **g**), 6.1 μm (length of the side of a square in 3D grid, **b**, **c**, **e**, **f**, **h**, **i**)

In WT tibiae, a small number of FABP4-immunopositive cells were sparsely observed along the COJ of the GP in the same layer as that occupied by numerous FABP5-immunopositive septoclasts in WT tibiae (Fig. 3a, d). In 3D-reconstruction, FABP4-immunopositive septoclasts were revealed to have several long processes (Fig. 3e, f) similar to FABP5-immunopositive cells in WT tibiae (Fig. 3b, c).

In FABP5^−/−^ tibiae, FABP4-immunopositive septoclasts were more numerous than those in WT-GP (Fig. 3d, g). In 3D-reconstruction, FABP4-immunopositive septoclasts in FABP5^−/−^ tibiae showed normal morphology as observed by FABP4- and FABP5-immunopositive septoclasts in WT tibiae (Fig. 3b, c, e, f, h and I, movies shown in Supplementary data S3, S4, S5). In morphometrical analysis, no significant difference in the length of processes of FABP4-positive septoclasts was detected between FABP5^−/−^ and WT tibiae (Fig. 3j).

### FABP4-expression in a partial population of FABP5-positive septoclasts

**Fig. 4:**
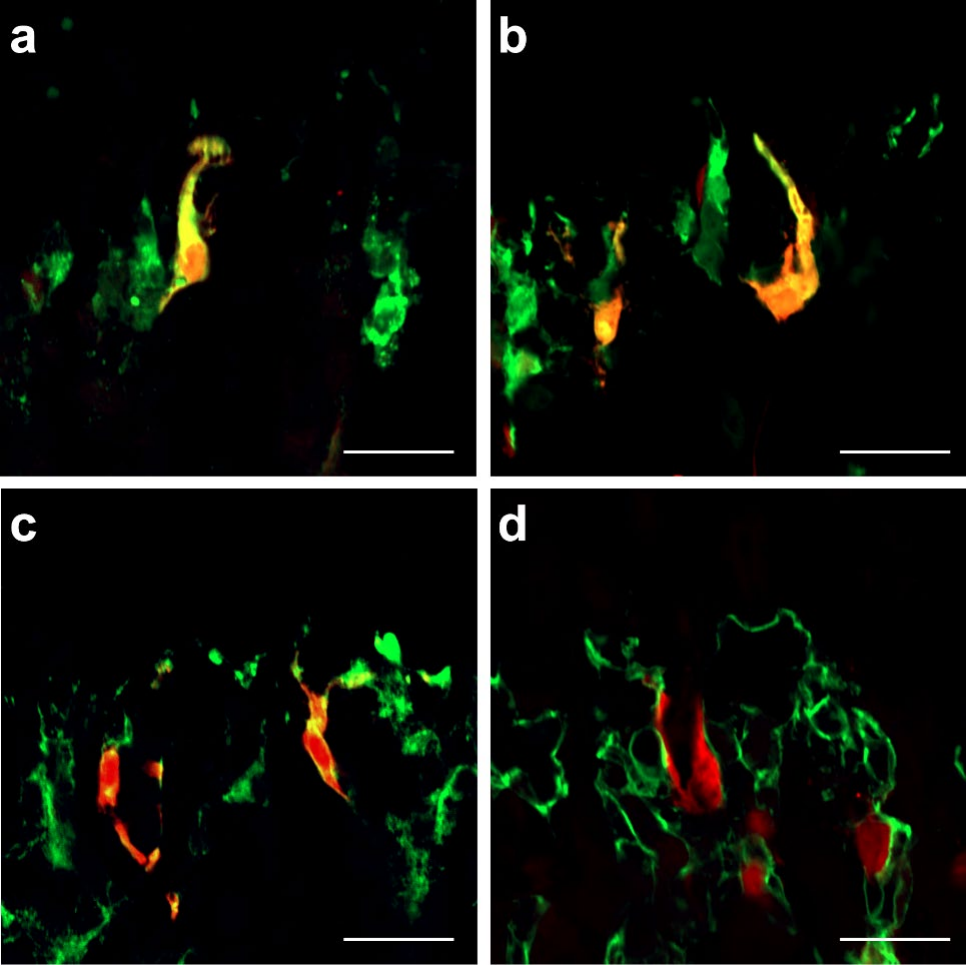
Expression of FABP4 in FABP5-immunopositive septoclasts in WT mice. Light micrographs of longitudinal sections at the chondro-osseous junction (COJ) of proximal tibiae of WT mice stained for FABP4 (red) plus cathepsin B (green) (**a**), for FABP4 (red) plus FABP5 (green) (**b**), for FABP4 (red) plus PDGFRβ (green) (**c**), and for FABP4 (red) plus CD31 (green) (**d**). Scale bars: 20 μm

In double-staining for either two of cathepsin B, FABP5 and PDGFRβ, a specific marker of pericyte (Lindahl et al. 1997; Winkler et al. 2010), immunoreactivities for all the three molecules were simultaneously localized in FABP4-positive cells (Fig. 4a–c). FABP4-positive cells were located adjacent to endothelial cells immunopositive for CD31, a marker of capillary sprouting (Albelda et al. 1991), in the same way as FABP5-positive septoclasts as previously reported by us (Bando et al. 2014) (Fig. 4d).

### PPARγ in FABP4-positive septoclasts in FABP5-/- tibiae

**Fig. 5:**
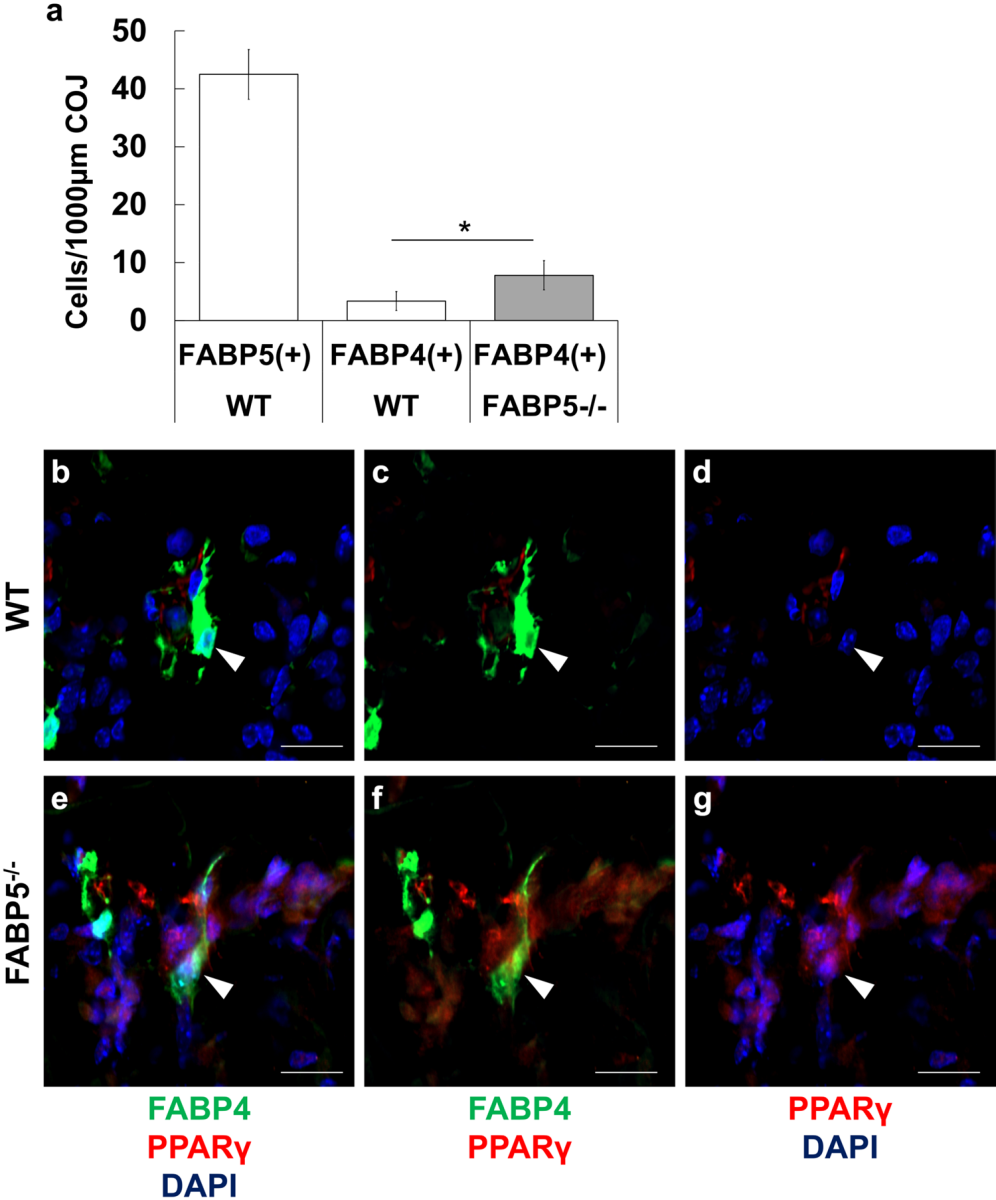
Cell count of FABP4-immunopositive septoclasts (**a**) and light micrographs of longitudinal sections stained for PPAR (red) plus FABP4 (green) with DAPI (blue) at the COJ of proximal tibiae of WT mice (**b**–**d**) and FABP5^−/−^ mice (**e**–**g**). Number of FABP5-immunopositive cells represents that of septoclasts (**a**). Arrowheads: lack (**b**–**d**) or occurrence (**e**–**g**) of immunoreactivity of PPAR in the nucleus of FABP4-positive septoclasts. **a** Mean SD, *P < 0.001 [*n* = 20]. Scale bars: 20 μm

In tibiae of WT mice aged 3 weeks, FABP4-immunoreactivity was detected in approximately 8% of a total population of FABP5-positive septoclasts in random sections (Fig. 5a). The population density of FABP4-immunopositive septoclasts in FABP5^−/−^ tibiae were approximately 2.5 times greater than that in WT tibiae (Fig. 5a).

To elucidate the mechanism of increase of FABP4-positive septoclasts, the expression of PPARγ which binds FABP4 and induces cell differentiation were examined (Lowell 1999; Hihi et al. 2002; Tan et al. 2002; Adida and Spener 2006) in FABP4-positive cells of FABP5^−/−^ and WT mice. Imunoreacitivity for PPARγ was detected in the nuclei of FABP4-immunopositive septoclasts of FABP5^−/−^ tibiae (Fig. 5e–g), in contrast to the lack of PPARγ-immunoreactivity in FABP4-positive septoclasts of WT tibiae (Fig. 5b–d).

### PPARγ in FABP4-positive septoclasts in GW1929-treated mice

**Fig. 6:**
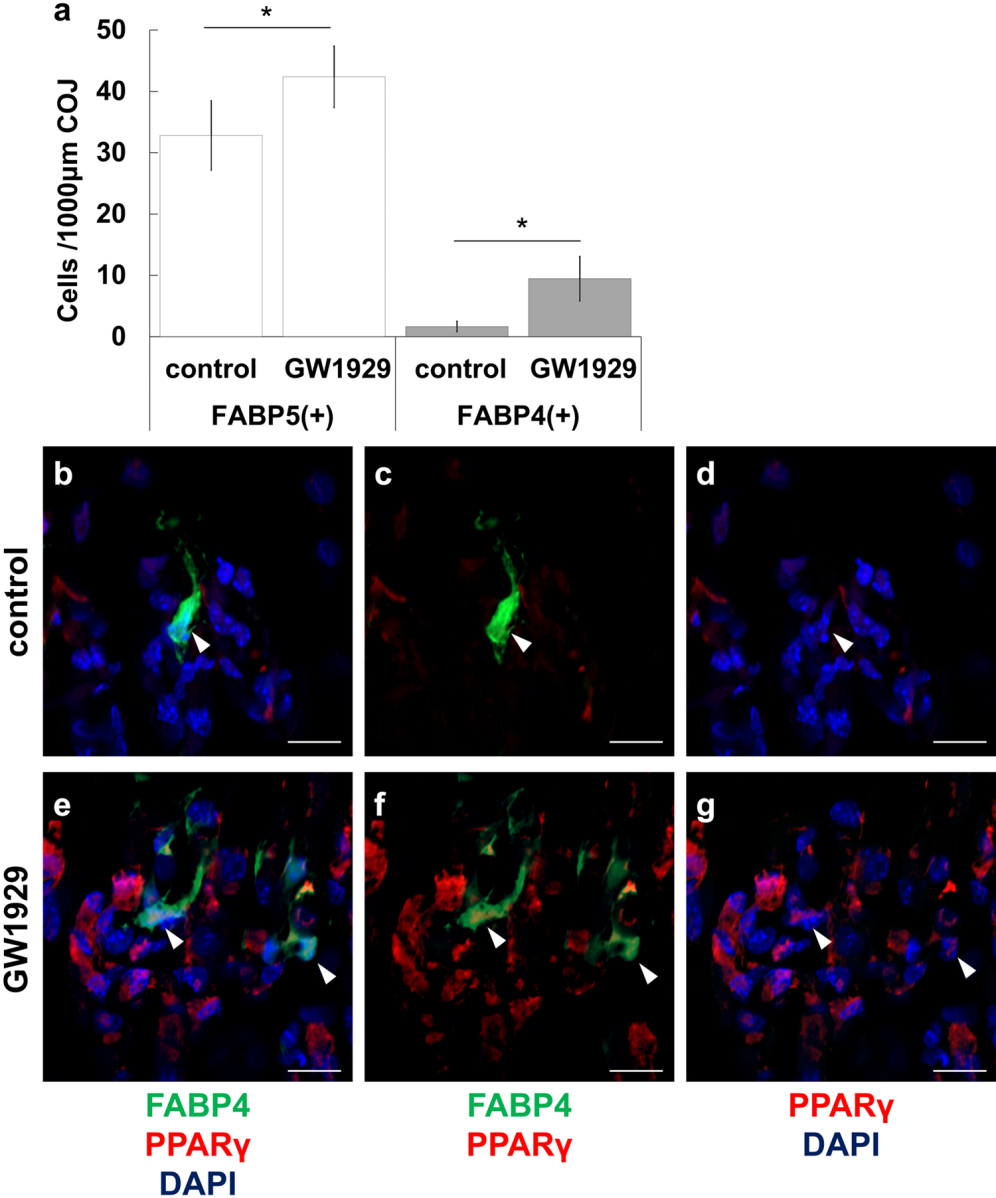
Cell count of FABP4-immunopositive septoclasts (**a**) and light micrographs of longitudinal sections stained for PPAR (red) plus FABP4 (green) with DAPI (blue) at the COJ of proximal tibiae of control mice (**b**–**d**) and PPAR agonist (GW1929)-treated mice (**e**–**g**). Number of FABP5-immunopositive cells represents the number of septoclasts (**a**). Arrowheads: lack (**b**–**d**) or occurrence (**e**–**g**) of immunoreactivity of PPAR in the nucleus of FABP4-positive septoclasts. **a** Mean SD, *P < 0.01 [*n* = 20]. Scale bars: 20 μm

FABP4-immunoreactivity was detected in 5% of FABP5-immunopositive septoclasts in tibiae of WT control mice aged 5 weeks (Fig. 6a). The population density of FABP4-positive septoclasts in tibiae of GW1929-treated mice was approximately 6 times greater than that in the control (Fig. 6a). By GW1929-administration, the population density of FABP5-immunopositive cells increased as that of FABP4-immunopositive cells increased (Fig. 6a). Although no expression of PPARγ was detected in FABP4-positive septoclasts of control tibiae (Fig. 6b–d), PPARγ occurred in the nucleus of FABP4-positive septoclasts in tibiae of the PPARγ agonist-treated mice (Fig. 6e–g).

## Discussion

### Cell shape and function of septoclasts mediated by FABP4 and FABP5

The shape, thickness and extension of septoclastic processes is correlated with the activity extent of the cartilage resorption. The septoclastic processes in cartilages under active resorption are longer than those in static or resting cartilages (Bando et al. 2014; 2018). The reduction in the GP cartilage resorption is induced by loose contact between transverse septa and morphologically abnormal septoclastic processes in tibiae of toothless rats (Gartland et al. 2009) and in tibiae of retinoic acid-excessive or deficient mice (Bando et al. 2017). Septoclasts in the present FABP5-deficiency mutant tibiae had shorter processes than those of WT control in a way similar to septoclasts in tibiae of vitamin A-deficiency mice, in which the activity of septoclasts to resorb cartilages is reduced (Bando et al.^2017^). This finding suggests that FABP5 is crucial for the cartilage resorption activity of septoclasts.

The present study revealed that FABP4 was newly expressed in a substantial number of septoclasts originally expressing FABP5 after its gene-deletion and that FABP4-positive septoclasts were morphologically normal. These results suggest that FABP4 can compensate the functional role of FABP5 in cartilage resorption in septoclasts.

### Functional significance of compensative increase in number of FABP4-positive septoclasts in FABP5-deficiency

During the resorption of hypertrophic cartilages of the GP, blood vessels invade the empty lacunae lacking. The compensation for the down-regulation of cartilage resorption by septoclasts may be carried out in FABP5-deficiency.

FABPs have redundant and overlapping roles in mediating uptake of fatty acids in　various tissues (Hotamisligil and Bernlohr 2015). FABP4 and FABP5 have 52% amino acid similarity and bind to several fatty acids with similar affinity and selectivity (Haunerland and Spener 2004; Furuhashi et al. 2014). Both FABP4 and FABP5 play a critical role in fatty acid uptake in capillary endothelial cells of heart and skeletal muscle (Iso et al. 2013). It is, therefore, possible that not only FABP5 but also FABP4 would contribute to the fatty acid transport into septoclasts. Since major FABPs (FABP1, 2, 3, 7) were not expressed in septoclasts in the present study, further studies are needed to look for other fatty acid- or lipid- binding proteins which have redundant or overlapping roles with FABP5 in septoclasts.

PPARs consist of three isotypes termed PPARα, β/δ, and γ, and they belong to the nuclear receptor superfamily and they are regulators of cell differentiation, tissue development, and energy metabolism (Brunmeir and Xu 2018). Specific pairs of FABP1/PPARα, FABP1/PPARγ, FABP4/PPAR and FABP5/PPARβ/δ have been reported to be involved in the signaling mechanisms (Schachtrup et al. 2008).

Cellular retinoic acid-binding protein (CRABP)-II has high affinity to RA in cytosol and deliver it to nuclear receptors (Napoli 2017). Retinoic acid receptor (RAR) whose isotypes (α, β and γ) are members of the superfamily of nuclear hormone receptors mediate transcriptional activation by RA (Chambon 1996). There has been evidence that cell survival/proliferation mediated by FABP5/PPARβ/δ or cell growth arrest/apoptosis mediated by CRABP-II and RAR is induced by retinoic acid signaling pathways and their alternate activation is determined by the FABP5/CRABP-II ratio (Schug et al. 2007). We previously reported that FABP5 and PPARβ/δ were expressed simultaneously in septoclasts, and cell proliferative or apoptotic effects was determined by the FABP5/CRABP-II ratio in septoclasts (Bando et al. 2017).

FABP4 has high affinity to PPARγ (Tan et al. 2002; Adida and Spener 2006). The present study showed that a lack of significant immunoreactivity for PPARγ was observed in septoclasts under normal physiological condition. However, both FABP4 and PPARγ were expressed simultaneously not only in septoclasts of GW1929, PPARγ agonist, -treated mice but also in those of FABP5^−/−^ mice. These findings indicate that the PPARγ signaling, resulting in cell proliferation/differentiation, is induced in FABP4-positive septoclasts in FABP5-deficiency. There has been evidence that differentiation from precursor cells is induced by the PPARγ signaling in adipocytes (Tontonoz et al. 1994; Chawla et al. 1994) and macrophages (Tontonoz et al. 1998; Pelton et al. 1999). The increase in population density of FABP4-positive septoclasts in FABP5-deficiency may represent the PPARγ signaling-induced differentiation of septoclasts from pericytes, their precursor cells (Bando et al. 2018).

Since increment of FABP4-positive septoclasts may be insufficient to compensate for the reduction in cartilage resorption activity of septoclasts in FABP5-deficiency, we speculate that other phagocytes such as osteoclasts (Feng and Teitelbaum 2013) or macrophages (Schlundt et al. 2018) may also support resorption of matrix of the GP cartilage. In rheumatoid arthritis, cartilage destruction is caused by degradation enzymes such as matrix metalloproteinase (MMP) produced by chondrocytes, synovial fibroblasts and macrophages and is associated with bone loss caused by increased resorption activity of osteoclasts (Tateiwa et al. 2019; Steffen et al. 2019). Further studies are needed to elucidate the compensation mechanisms for the reduction of cartilage resorption activity of septoclasts in FABP5-deficiency.

Altogether, the present study suggests that a pair of FABP5 and PPARβ/δ is constitutively expressed in septoclasts and contribute to survival/proliferative effects of septoclasts, while a pair of FABP4 and PPARγ is newly induced in septoclasts under FABP5-deficiency, and compensates for resulting reduction of cartilage resorption activity.

## Supplementary Information

Below is the link to the electronic supplementary material.

Supplementary file1 (AVI 456454 KB)

Supplementary file2 (AVI 456454 KB)

Supplementary file3 (AVI 456454 KB)

Supplementary file4 (AVI 456454 KB)

Supplementary file5 (AVI 456454 KB)
